# Age and gender disparities in oral anticoagulant use: a nine-year nationwide drug utilization analysis

**DOI:** 10.3389/fphar.2026.1770826

**Published:** 2026-02-12

**Authors:** Ahmed A. A. Omer, Márta Csatordai, Réka Viola, Péter Doró

**Affiliations:** 1 Institute of Clinical Pharmacy, Faculty of Pharmacy, University of Szeged, Szeged, Hungary; 2 Department of Pharmacology, Faculty of Pharmacy, University of Gezira, Wad Madani, Sudan; 3 Institute of Clinical Pharmacy, Albert Szent-Györgyi Health Centre, University of Szeged, Szeged, Hungary

**Keywords:** age, direct oral anticoagulants, drug utilization, gender, Hungary, oral anticoagulants, vitamin K antagonists

## Abstract

**Background:**

Cardiovascular diseases remain the leading cause of mortality in Europe, responsible for one-third of all deaths in 2021. In Hungary, the standardized death rate from cardiovascular diseases reached 722.8 per 100,000 individuals, more than double the EU average. Oral anticoagulants (OACs) play a crucial role in lowering cardiovascular mortality. The use of OACs has shifted rapidly over the past decade with the transition from vitamin K antagonists (VKAs) to direct oral anticoagulants (DOACs). Although previous studies have examined gender differences in OACs prescribing, most rely on patient-level registries and do not capture population-level exposure.

**Aim:**

To evaluate population-level trends in OACs consumption in Hungary from 2014 to 2022, stratified by age and gender, with a specific focus on detecting temporal changes in the gender gap.

**Method:**

National level data were obtained from the Hungarian National Health Insurance Fund database, which includes all reimbursed prescription medications for the entire population. Utilization was measured using the WHO’s ATC/DDD methodology and expressed as defined daily doses per 1,000 inhabitants per day (DID). Linear regression was used to assess trends.

**Results:**

Total OACs use nearly doubled over the study period, increasing from 9.79 to 18.73 DID (Coeff. = 1.18, p < 0.001). VKAs use declined significantly in both genders by 41.2% in males and 47.8% in females, while DOACs use increased more than tenfold. OACs utilization was consistently higher in males. Gender differences in DOACs, initially negligible, began to widen from 2018 onward, whereas, VKAs utilization exhibited a consistently wider gender difference across all years. OACs use increased with age, peaking in the 80–84 age group. Apixaban became the most used OAC, reaching 6.11 DID in 2022.

**Conclusion:**

A marked shift from VKAs to DOACs occurred, with overall OACs use nearly doubling, with a widening gender gap in consumption of both VKAs and DOACs. Utilization was consistently higher among males and increased with age, highlighting the importance of continued monitoring to ensure effective and evidence-based anticoagulant use.

## 1 Introduction

Circulatory diseases are a leading cause of mortality in the European Union (EU), accounting for 32.4% of all deaths in 2021, followed by cancer (21.6%) and COVID-19 (10.7%). The standardized death rate from circulatory system diseases across European countries is 343.4 per 100,000 inhabitants, but in Hungary, this rate is more than double, reaching 722.8 per 100,000 (Eurostat, 2025). Among circulatory diseases, venous thromboembolism (VTE), which includes deep vein thrombosis (DVT) and pulmonary embolism (PE), represents the third most common cause of vascular death after myocardial infarction and stroke (Stančiaková et al., 2024).

Oral anticoagulants (OACs) play crucial roles in the prevention and treatment of thromboembolism, as well as in stroke prevention, for patients with nonvalvular atrial fibrillation (NVAF). Traditionally, vitamin K antagonists (VKAs), such as warfarin, have been the cornerstone of anticoagulation therapy. However, their use has been limited by the need for frequent monitoring, dietary restrictions, drug interactions, and a narrow therapeutic window (Martins et al., 2025; Omer et al., 2024; Tan and Lee, 2021). Direct oral anticoagulants (DOACs), after their approval, have become leading therapeutic substitutes for VKAs in providing convenient, effective, safe treatment and prevention choices for patients with different thromboembolic disorders; stroke prevention in NVAF; and treatment of DVT and PE (Choumane et al., 2018). Over the last decade, their clinical applications have broadened considerably, extending from cardiovascular indications to include prophylaxis of VTE in patients undergoing orthopedic surgery, emphasizing their growing importance in routine clinical practice (Turpie et al., 2009; Beyer-Westendorf et al., 2017; Fuji et al., 2014; Lassen et al., 2010a; Lassen et al., 2010b). The transition from VKAs to DOACs has been observed globally and is influenced by clinical trial evidence, updated guideline recommendations, and healthcare policy changes (Van Gorp and Schurgers, 2015; January et al., 2019).

OACs use continues to rise globally. In the United States, the proportion of patients with atrial fibrillation (AF) receiving OACs has increased, driven largely by a shift from VKAs to DOACs (Navar et al., 2022). Similar trends have been reported in large international cohort studies across the United States, United Kingdom, and Europe, where DOACs have become the preferred initial therapy (Vora et al., 2022). Hungary has demonstrated the same transition in real-world practice. Kazareczki et al. (2024) showed that by 2020, DOACs had overtaken VKAs as the predominant anticoagulant among patients undergoing catheter ablation for AF, reflecting evolving guidelines and prescribing practices (Letícia et al., 2024). A nationwide analysis using the Hungarian Health Insurance Fund database further reported a 28% reduction in 3-year mortality among AF patients treated with DOACs compared with VKAs, with the greatest benefit in younger patients and those at lower thromboembolic risk. The survival advantage of DOACs was most pronounced in the early treatment period, with a 33% reduction in mortality within the first 3 months (Papp et al., 2023). These findings provide compelling real-world evidence for the clinical advantages of DOACs and reinforce the need to understand long-term utilization trends at the population level. Despite increasing attention to gender differences in OACs use, most existing evidence is derived from patient-level registries that assess initiation, prescription rate, or persistence (Hirschfeld et al., 2023; Sur et al., 2019; Thompson et al., 2017; Yong et al., 2020; Lee et al., 2023; Dalmau et al., 2021). Even population-based studies, such as the Finnish FinACAF analysis, assessed only OACs initiation rates and did not evaluate overall consumption (Teppo et al., 2024). To date, no study has analyzed gender- and age-specific trends in OACs use, focusing on both VKAs and DOACs, using the World Health Organization Anatomical Therapeutic Classification/Defined Daily Dose system (WHO ATC/DDD). This perspective may identify disparities that are not visible on patient-level studies. Furthermore, despite the growing evidence supporting DOACs use, national-level data on OACs utilization by age group and gender remain unexplored in Hungary. Examining these patterns can provide important insights into the implementation of guideline recommendations, the impact of healthcare policy changes, and potential disparities in OACs use. The aim of this study to evaluate population-level trends in OACs consumption in Hungary from 2014 to 2022, stratified by age and gender, with a specific focus on detecting temporal changes in the gender differences both in VKAs and DOACs use.

## 2 Methods

### 2.1 Study design and data source

A retrospective analysis of drug utilization was performed, covering the period between 2014 and 2022. The data originated from the medication dispensing database of the Hungarian National Health Insurance Fund, the sole and mandatory health insurance provider in Hungary. This database contains utilization data on each reimbursed medication for the entire population of Hungary (9.6 million in 2022) (Hungarian Central Statistical Office, 2025). Aggregated utilization data were calculated and stratified by calendar year, gender and age category (5 years). Age- and gender-stratified population data were derived from the database of the Hungarian Central Statistical Office (Hungarian Central Statistical Office, 2025). The following variables were included: calendar year, active ingredient, strength, package size, number of packages, ATC code, DDD per package, gender, and age category.

### 2.2 Drug classification and inclusion criteria

The data were analysed according to the WHO ATC/DDD (version 2024), and the filtered ATC code was B01, which corresponds to antithrombotic agents. Drug consumption was expressed as the defined daily dose per thousand inhabitants per day (DID) (WHO Collaborating Centre for Drug Statistics Methodology, 2024). The DDD is the assumed average daily maintenance dose of the medication used for its main indication in adults. The DID was calculated via the following formula: (total number of DDDs used in 1 year × 1,000)/(population × 365) (World Health Organization, 2003).

For the present study, we analysed the following oral anticoagulants: VKAs (ATC: B01AA), direct thrombin inhibitors (ATC: B01AE), and direct factor Xa inhibitors (ATC: B01AF). Antiplatelet agents and parenteral anticoagulants were excluded from this analysis. For the antithrombotic drug groups, the following drugs were included: warfarin (B01AA03), acenocumarol (B01AA07), dabigatran etexilate (B01AE07), rivaroxaban (B01AF01), apixaban (B01AF02), and edoxaban (B01AF03).

### 2.3 Statistical analysis

To analyse time trajectories in the utilization of OAC drug groups, simple linear regression analysis was performed separately for each gender and age stratum. The results were summarized via the regression coefficient and its corresponding p value, with statistical significance defined as P < 0.05. The dependent variable was the use of OAC drugs measured in DID, whereas the independent variable was time (in years). The regression coefficient reflects the trend direction and describes the average annual changes in medication use. A positive coefficient indicates an increasing trend, whereas a negative coefficient signifies a decreasing trend.

Additionally, descriptive statistics were used to describe the sum of yearly medication use, and relative use was expressed as the proportion of total OAC drug use. Microsoft Excel (Microsoft Office Professional Plus 2019; Microsoft Corp., Redmond, WA, United States) and R software (version 4.4.2, Pile of Leaves; R Foundation for Statistical Computing, Vienna, Austria) were used.

### 2.4 Ethical approval

This study used only aggregated, non-identifiable drug utilization data and did not involve access to any individual patient-level information; therefore, ethical approval was not required.

## 3 Results

### 3.1 Utilization trends of OACs

During the 9-year study period, overall OACs use nearly doubled, increasing by 91.2%, to 18.73 DID in 2022. VKAs use decreased to 4.90 DID, whereas DOACs use increased significantly, reaching 13.83 DID (Table 1).

**TABLE 1:** OACs utilization in Hungary (DOACs and VKAs) between 2014 and 2022 expressed as defined daily dose per thousand inhabitants per day (DID) and as a percentage of total use.

Drug class	2014	2015	2016	2017	2018	2019	2020	2021	2022	% Change	Coeff	P value
DOACs	0.99 (10.11%)	1.88 (17.78%)	2.95 (25.69%)	4.66 (36.54%)	6.54 (46.20%)	8.52 (54.76%)	10.55 (61.68%)	12.07 (68.31%)	13.83 (73.85%)	1,296.3	1.68	<0.001
VKAs	8.80 (89.89%)	8.70 (82.22%)	8.52 (74.31%)	8.08 (63.46%)	7.62 (53.80%)	7.04 (45.24%)	6.56 (38.32%)	5.60 (31.69%)	4.90 (26.15%)	−44.3	−0.50	<0.001
Total (OAC)	9.79	10.58	11.47	12.74	14.16	15.56	17.11	17.66	18.73	91.2	1.19	<0.001

DOAC, direct oral anticoagulant; OAC, oral anticoagulant; VKA, vitamin K antagonist.

### 3.2 The consumption of different OAC drugs

Warfarin utilization was relatively stable, with a slight declining trend: 2.58 DID in 2014 increased to a peak of 3.24 DID in 2018–2019, before declining to 2.49 DID in 2022. In 2014, acenocumarol was used most frequently, but since 2015, its use has dynamically decreased to 2.41 DID in 2022 (Table 2). Among DOACs, apixaban was the most commonly used DOAC with 6.11 DID, followed by rivaroxaban (4.22), dabigatran etexilates (1.99), and finally edoxaban, which was introduced in 2017, reaching a 1.50 DID in 2022 (Table 2).

**TABLE 2:** Utilization of different OACs in Hungary between 2014 and 2022 expressed as defined daily dose per thousand inhabitants per day (DID).

Drug	ATC code	2014	2015	2016	2017	2018	2019	2020	2021	2022	% Change	Coeff	P value
Warfarin	B01AA03	2.58	2.84	3.07	3.16	3.24	3.24	3.15	2.76	2.49	−3.4	−0.01	<0.887
Acenocumarol	B01AA07	6.23	5.86	5.45	4.93	4.37	3.80	3.41	2.84	2.41	−61.3	−0.49	<0.001
Dabigatran etexilate	B01AE07	0.25	0.50	0.72	1.11	1.40	1.63	1.87	1.94	1.99	697.2	0.24	<0.001
Rivaroxaban	B01AF01	0.71	1.17	1.72	2.43	2.99	3.42	3.80	3.98	4.22	495.0	0.46	<0.001
Apixaban	B01AF02	0.03	0.21	0.51	1.07	1.76	2.70	3.76	4.82	6.11	>1,000%^a^	0.77	<0.001
Edoxaban	B01AF03	-	-	0.00	0.04	0.39	0.77	1.12	1.33	1.50	>1,000%^b^	0.22	<0.001

^a^ Apparent extreme increases due to near-zero baseline in 2014 (0.03 DID).

^b^ Edoxaban was introduced after 2015; extremely high % change reflects uptake from 0.00 DID, in 2016.

### 3.3 Gender-specific trends in DOACs utilization

**FIGURE 1:**
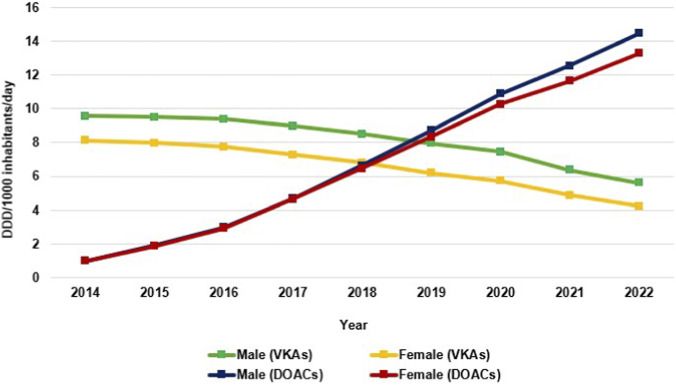
Utilization of VKAs and DOACs among males and females between 2014 and 2022 expressed as defined daily dose per thousand inhabitants per day (DID).

DOACs consumption increased significantly in both genders, with slightly higher use in males, increasing from 0.99 DID in 2014 to 14.4 DID in 2022 (coefficient = 1.76, p value < 0.001). In females, consumption increased from 0.99 DID in 2014 to 13.2 DID in 2022 (coefficient = 1.161, p value < 0.001) (Figure 1; Supplementary Table S1). DOACs use increased similarly in males and females during the early study years (2014–2017), gender differences were minimal, with absolute gaps below 0.06 DID throughout this period. A noticeable divergence emerged beginning in 2018, when male consumption exceeded female consumption by 0.20 DID, followed by a progressive annual widening. By 2022, the gender difference reached 1.17 DID, representing the largest observed gap (Supplementary Table S1). This corresponds to a widening gap difference from 0.8% in 2014 to 8.9% in 2022 (Supplementary Figure S3).

### 3.4 Utilization trends of DOACs across age categories over time

**FIGURE 2:**
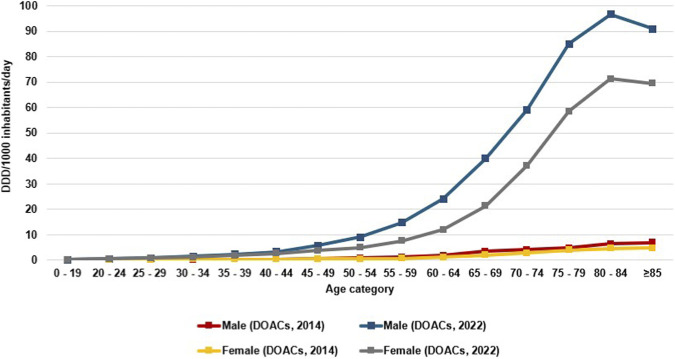
Utilization of DOACs among males and females across different age categories in 2014 and 2022 expressed as defined daily dose per thousand inhabitants per day (DID).

DOACs utilization consistently and gradually increased among all age groups of both genders (Supplementary Table S2; Supplementary Figure S1). The use among the youngest individuals (0–19 years) was marginal for males and females. The maximum consumption was found in the 80–84 age category; in males, it ranged from 6.31 DID in 2014 to 96.73 DID in 2022 (Coeff. 11.77, p value < 0.001), and female from 4.52 DID in 2014 to 71.23 DID in 2022 (Coeff. 8.79, p value < 0.001) (Figure 2; Supplementary Table S2).

### 3.5 Gender-specific trends in VKAs utilization

VKAs use decreased in both genders: by 41.2% in males (coefficient = - 0.50, p < 0.001) and by 47.9% in females (coefficient = - 0.50, p < 0.001) between 2014 and 2022. Consumption among males remained higher than among females (9.55 vs. 8.12 DID in 2014 and 5.61 vs. 4.23 DID in 2022, Figure 1). Males consistently exhibited higher VKAs consumption throughout the study period (Supplementary Table S3), and the gender difference in VKAs utilization gradually increased throughout the study period, from 17.7% in 2014 to 32.6% in 2022 (Supplementary Figure S3).

### 3.6 Utilization trends of VKAs across age categories over time

**FIGURE 3:**
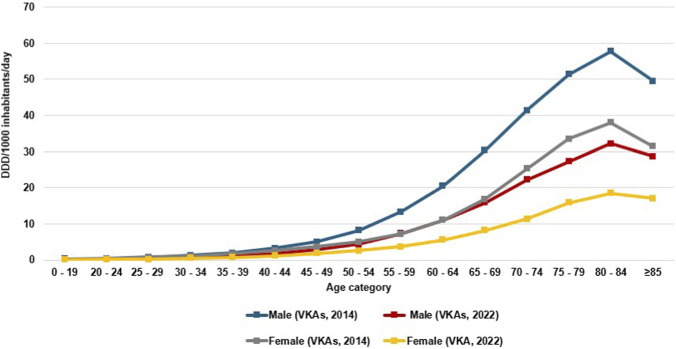
Utilization of VKAs among males and females. across different age categories in 2014 and 2022 expressed as defined daily dose per thousand inhabitants per day (DID).

VKAs utilization gradually decreased across all age categories in both genders (Supplementary Table S4; Supplementary Figure S2). The utilization among the youngest age group (0–19 years) remained very low throughout the study. The highest utilization occurred in the 80–84 years age group; 57.7 DID in males in 2014, 32.2 DID in 2022 (coefficient = −3.21, p value < 0.001), and 37.9 DID in female in 2014, 18.5 DID in 2022 (coefficient = −2.57, p value < 0.001) (Figure 3; Supplementary Table S4).

## 4 Discussion

This study represents the first population-based analysis of OACs utilization trends in Hungary, by age and gender, over a 9-year period. Overall OACs use increased substantially, driven by the transition from VKAs to DOACs, which is consistent with trends observed in other European countries. DOACs consumption increased significantly across the study period, while VKAs use declined. However, this analysis using WHO ATC/DDD methodology capturing total drug exposure, revealed a distinct and previously unreported pattern: a significant widening gender gap in DOACs consumption. Although male and female DOACs use was nearly identical during the early adoption years of the study, a clear divergence emerged from 2018 onward, with males exhibiting a significantly steeper rise in exposure, whereas, VKAs utilization exhibited a consistently wider gender difference across all years.

The greater use of OACs among males than females in this study aligns with many studies (Teppo et al., 2024; Mitchell et al., 2021; Xia et al., 2022; Ka et al., 2017). Evidence consistently shows that VTE, a major indication for OACs, recurs more frequently in men; for example, the PROLONG study demonstrated greater recurrence in males (Cosmi et al., 2010). A meta-analysis of seven studies also reported greater recurrence in men at both 1 year (9.5% vs. 5.3%) and 3 years (11.3% vs. 7.3%) (Douketis et al., 2011). An analysis combining four European cohorts (CARROT, CVTE, AUREC, and LETS follow-up) reported that men had a 2.8 fold greater risk of recurrence (Roach et al., 2015). A multinational cohort study from nine databases also found that males represented a greater proportion of OACs users among AF patients (Vora et al., 2022). Moreover, many studies have highlighted gender differences in anticoagulant prescribing patterns; although women often have higher CHADS_2_ scores, they are less likely to receive anticoagulation (Teppo et al., 2024; Ka et al., 2017; Giner-Soriano et al., 2023). For example, one study reported that, compared with 82.5% of men, only 76.8% of women were prescribed anticoagulants (p < 0.001), with this gap being more pronounced among patients over 75 years of age, possibly because of clinicians’ concerns about increased bleeding risk in elderly women (Ka et al., 2017). Similar patterns have been reported in the South Korean CODE-AF registry, where women more often receive suboptimal DOACs doses (Lee et al., 2018). Similarly, a report from the PINNACLE and GLORIA-AF registries revealed that women were less likely than men to receive OACs (Thompson et al., 2017; Mazurek et al., 2018).

The markedly lower use of VKAs among females than males in this study may reflect evidence showing that females with AF treated with warfarin have a higher residual risk of stroke or systemic embolism than males, with odds ratios reported between 1.2 and 2.9 in various studies (Pancholy et al., 2014; Poli et al., 2009). Therefore, women with NVAF are significantly less likely to be prescribed VKAs (Lee et al., 2023). In contrast, DOACs therapy appears to eliminate this disparity, with comparable rates of stroke and systemic embolism between gender (Pancholy et al., 2014). These outcome differences likely influence prescribing practices, leading to a greater reluctance to use VKAs in women due to their less favourable risk-benefit profile. However, it is important to note that DID reflects dispensed drug volume rather than treatment appropriateness, therefore, gender differences observed in our study may also be influenced by differences in dosing, persistence or discontinuation patterns, differential survival and health seeking behavior. These factors may contribute to higher aggregate OAC volumes in men without necessarily indicating inequitable prescribing.

The increase in OACs use cannot be explained by the emergence of DOACs alone. Hungary has one of the highest cardiovascular mortality rates in Europe, with circulatory diseases exceeding twice the EU average and VTE representing a major contributor to vascular deaths (Eurostat, 2025). This substantial disease burden has expanded the pool of patients requiring anticoagulation. Concurrently, European and national guidelines have broadened OACs indications, and increased awareness among clinicians and patients along with the clinical advantages of DOACs has further facilitated uptake. The availability of specific reversal agents has also likely strengthened prescriber confidence. Idarucizumab was approved in 2015 for dabigatran reversal and andexanet alfa in 2019 for factor Xa inhibitors (European Medicines Agency, 2025a; U.S. Food and Drug Administration (FDA), 2025; European Medicines Agency, 2025b). The availability of these specific antidotes may have provided reassurance to prescribers that DOACs effects could be promptly reversed in emergencies.

The increasing trend in OACs utilization, particularly the substantial shift from VKAs to DOACs, is not only unique to Hungary and mirrors broader European patterns (Vora et al., 2022; Lee et al., 2023; Zhu et al., 2018; Grymonprez et al., 2023; Alcusky et al., 2019; Forslund et al., 2018). For example, Austrian insurance data revealed d a 43% increase in OACs users between 2011 and 2014, driven by a nearly fivefold increase in DOACs prescriptions, whereas VKAs use increased only marginally (Schuh et al., 2016). In Belgium, OACs use among patients with AF increased markedly between 2013 and 2019, alongside a transition from VKAs to DOACs (Grymonprez et al., 2023). Danish national data similarly demonstrated more than double increase in OACs consumption (from 13.2 in 2014 to 27.5 DID in 2022) and exceeded consumption in Hungary by one-third in 2022 (18.73 DID vs. 27.5 DID) (The Danish Health Data Authority, 2025).

Apixaban emerged as the most frequently used DOACs by 2022, although it appeared on the Hungarian market in 2014. Despite rivaroxaban use exceeded that of apixaban through 2020, apixaban consumption increased remarkably in the final 2 years of the study.

Several studies have shown that apixaban has become the most used OACs for patients with NVAF in recent years. An analysis of data from over three million patients across the United States and Europe reported that, by 2017, apixaban was the leading initial OACs in the United States, United Kingdom, France, Germany, and Belgium, followed by rivaroxaban (Vora et al., 2022). Similarly, a major U.S. health plan dataset revealed that apixaban accounted more than half of all DOACs prescriptions by early 2017 (Zhu et al., 2018).

The higher utilization of apixaban than of other DOACs can be explained by several clinical advantages. Although all DOACs are FDA-approved for preventing stroke and systemic embolism in NVAF and for treating or preventing DVT and PE, apixaban is particularly suitable for patients with comorbidities (Chen et al., 2020). According to the 2018 AF guidelines, apixaban and warfarin are recommended for patients with end-stage renal disease (ESRD), but warfarin has little proven benefit and significant risk in this population, thus providing apixaban superiority for those patient cohorts (January et al., 2019; St et al., 2018). Moreover, apixaban requires no dose adjustment for renal impairment, including ESRD and hemodialysis, which increases its usability (Chen et al., 2020; Byon et al., 2019). It also offers dosing flexibility for special populations and is used cautiously in obese and low-body-weight patients (Chen et al., 2020). These characteristics make apixaban a preferred option across diverse clinical profiles, contributing to its leading utilization.

In this analysis, OACs utilization significantly increased with age, reflecting the well-established association between aging and increased VTE risk. Incidence rates increase exponentially across the lifespan from negligible levels in children to 450–600 per 100,000 person-years in individuals over 80 years of age (White, 2003). The LITE study further highlights this age gradient, reporting race- and gender-adjusted incidence rates per 1,000 person-years that increase from 0.72 (40–54 years) to nearly 7.0 in those aged ≥85 years (Lutsey and Zakai, 2023; Cushman et al., 2004). This higher risk among older adults is likely driven by the increased prevalence of risk factors such as obesity, cancer, hospitalization, and multiple comorbidities (Lutsey and Zakai, 2023; Kannel, 2005).

OACs utilization increased with age, peaking between 80 and 84 years. Similar patterns have been reported internationally. A large United Kingdom cohort reported the steepest initiation rates in patients aged 75–84 years, with rates declining beyond age 85 (Mitchell et al., 2021). In China, patients aged 85–89 and ≥90 years had significantly higher odds of not receiving OACs therapy than did those aged 80–84 years (Xia et al., 2022). Danish national registry data likewise demonstrated increasing OACs use with age in primary care (The Danish Health Data Authority, 2025).

Nevertheless, age-related comorbidities can also contribute to the underuse of OACs in the oldest age groups. Dementia reduces the likelihood of anticoagulation by 34%, whereas a history of falls or fractures reduces OACs use by 17% and 12%, respectively (Mitchell et al., 2021). Concomitant medications such as SSRIs or anticonvulsants may further decrease OACs prescribing (Mitchell et al., 2021). Geriatric syndromes, including frailty and malnutrition further influence prescribing decisions and are known predictors of rehospitalization and mortality in older adults (Sadıç et al., 2025).

Changes in Hungary’s age structure over the study period contributes to the overall increase in the use of OACs, since their use increases markedly with advancing age as our findings show. Moreover, our analyses report not only overall DID values, but DID values stratified by age groups, which partially mitigates denominator effects and allows trends within age strata to be examined directly.

This study has several strengths. Its stratified analysis provides detailed insights into age and gender specific OACs utilization, an approach not previously reported in Hungary. Second, the 9-year study period enabled the tracking of market entry and the progressive uptake of OACs. The use of WHO ATC/DDD methodology allowed for population-level comparisons of aggregated drug exposure, offering a complementary perspective to patient-level registry studies. With respect to the limitations of this study, although DID allows for the comparison of aggregated medication use across populations, it may differ from the actual prescribed daily dose, particularly for DOACs with dose adjustments (based on renal function, age, body weight) and gender specific dosing variations which may bias the comparison (World Health Organization, 2003). Importantly, this retrospective study focused on population-level utilization patterns and did not evaluate the appropriateness of individual treatment decisions. Finally, changes in the age structure of the Hungarian population during the study period may have influenced the observed trends.

## 5 Conclusion

This study demonstrated that OACs utilization in Hungary doubled between 2014 and 2022, driven by a marked increase in DOACs use and a corresponding decline in VKAs consumption, with apixaban becoming the most utilized OAC. The consumption of OACs gradually increased with age, with maximum utilization in the 80–84 years in both genders, after which it started to decline. The utilization of OACs was greater in males across the study period, and the results highlight an emerging gender imbalance both in VKAs and DOACs exposure and emphasize the need to explore clinical, behavioral, and system-level factors that may disproportionately affect long-term anticoagulation in females. These findings highlight evolving prescribing practices and the need for continued evaluation to ensure effective and evidence-based anticoagulant use in all age groups and in both genders.

## Data Availability

The data analyzed in this study is subject to the following licenses/restrictions: The datasets analyzed in this study are aggregated national drug utilization data that is not publicly available. Access to the data is subject to institutional and regulatory restrictions and therefore cannot be made publicly available. The data may be obtained from the corresponding author upon reasonable request. Requests to access these datasets should be directed to Péter Doró, Institute of Clinical Pharmacy, Faculty of Pharmacy, University of Szeged, 6725 Szeged, Hungary Email: doro.peter@szte.hu.
