# Supplementary material for: Age and gender disparities in oral anticoagulant use: a nine-year nationwide drug utilization analysis
Source: Front Pharmacol. 2026 Feb 12;17:1770826. doi: 10.3389/fphar.2026.1770826 (PMC12935904; doi:10.3389/fphar.2026.1770826)
Supplement: Supplementary file 1 [file DataSheet1.pdf]

# *Supplementary Material*

## 1 Supplementary Figures and Tables

### 1.1 Supplementary Figures

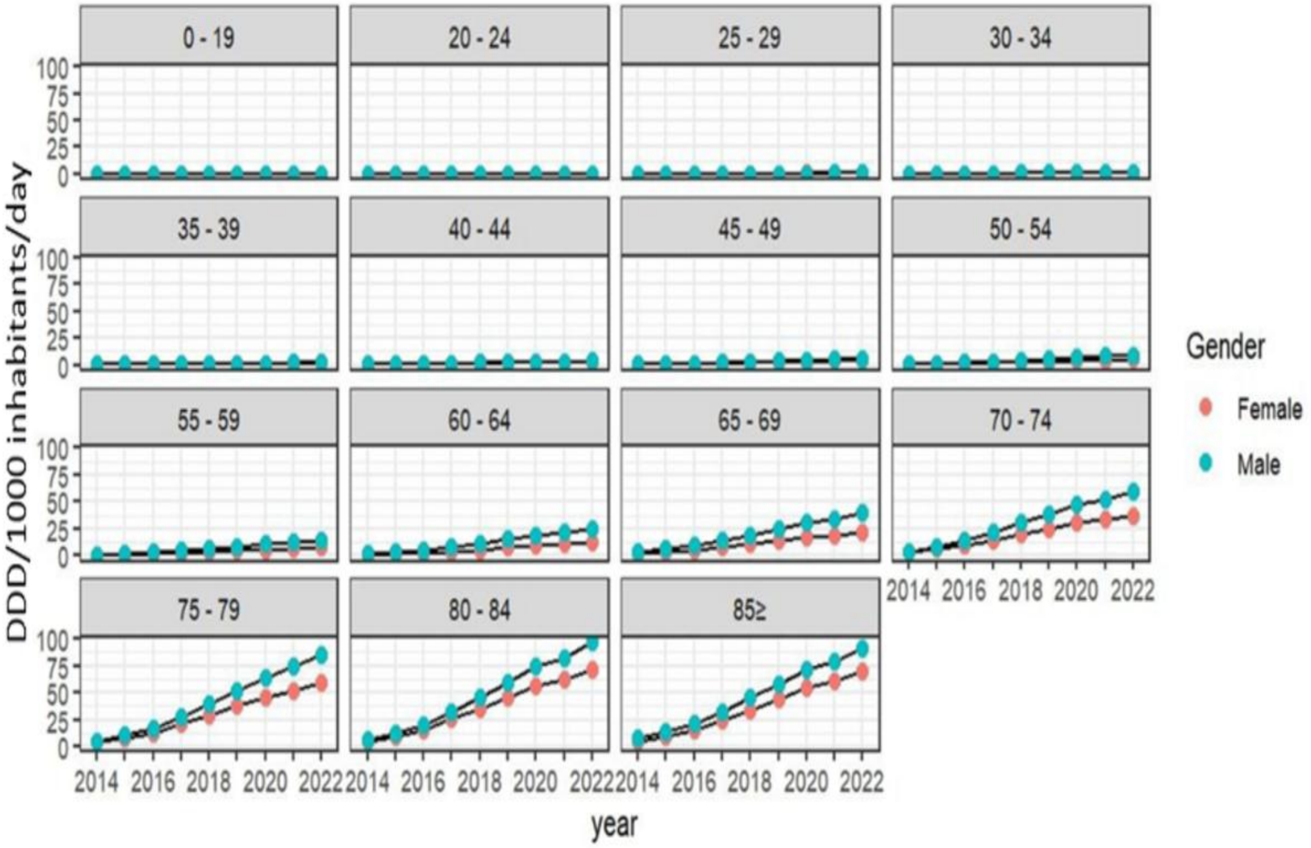


**Figure S1.** Utilization of DOACs among males and females in different age groups from 2014 and 2022 expressed as defined daily dose per thousand inhabitants per day (DID)

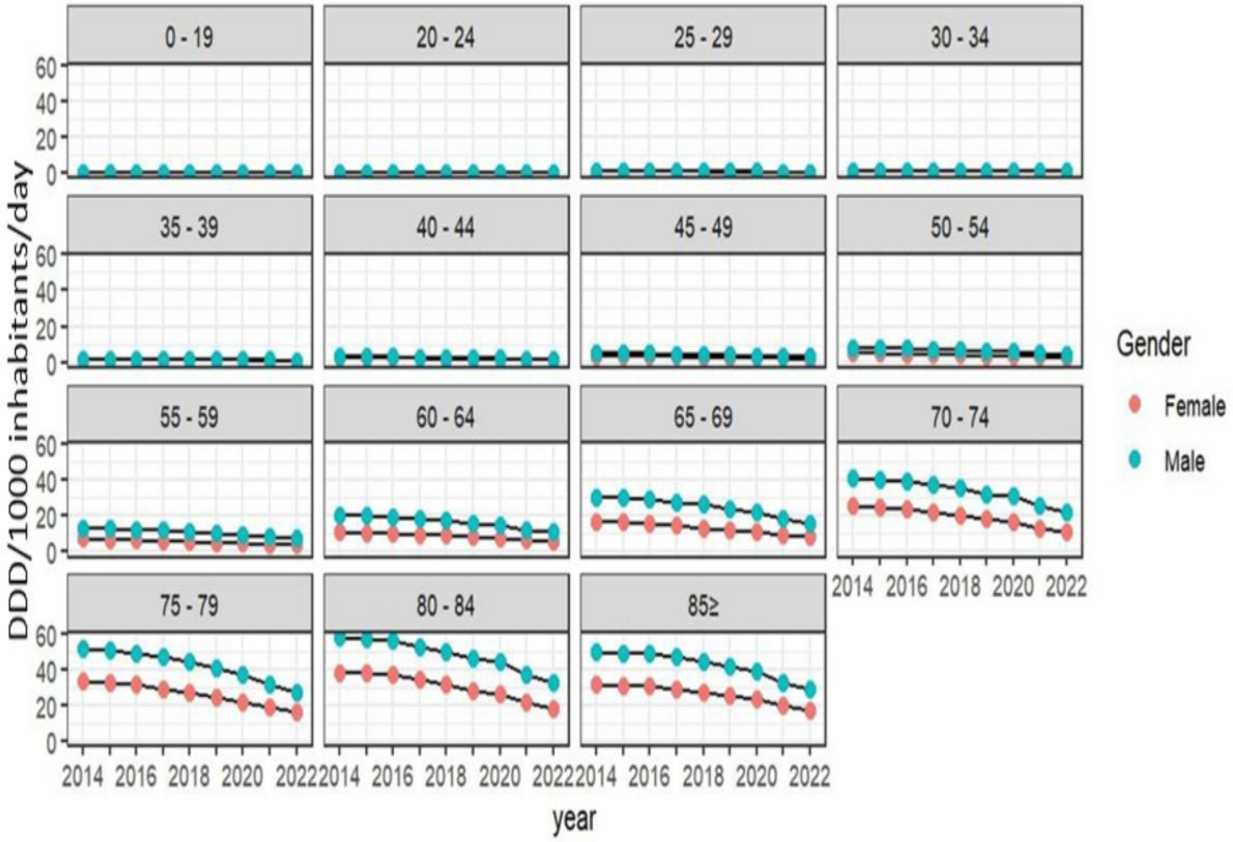


**Figure S2.** Utilization trends of VKAs among males and females in Hungary between 2014 and 2022 expressed as defined daily dose per thousand inhabitants per day (DID)

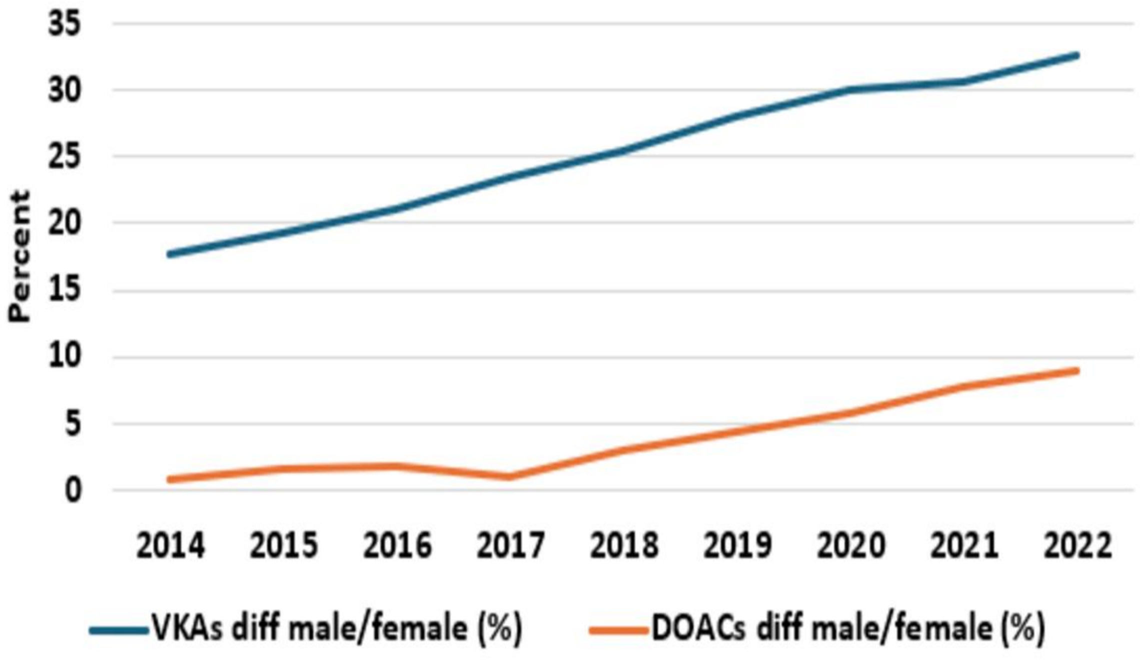


**Figure S3**. Percentage of gender difference of VKAs and DOACs utilization from 2014 and 2022 calculated as ((male (DID)/female (DID))-1) *100

## 1.2 Supplementary Tables

**Table S1** Yearly DOACs consumption by gender in Hungary, 2014 - 2022 (DID):

| Year | Male (DID) | Female (DID) | Difference (Male - Female) |
|---|---|---|---|
| 2014 | 0.99 | 0.99 | 0.00 |
| 2015 | 1.90 | 1.87 | 0.03 |
| 2016 | 2.97 | 2.92 | 0.05 |
| 2017 | 4.68 | 4.63 | 0.05 |
| 2018 | 6.64 | 6.45 | 0.19 |
| 2019 | 8.71 | 8.34 | 0.37 |
| 2020 | 10.86 | 10.27 | 0.59 |
| 2021 | 12.54 | 11.63 | 0.91 |
| 2022 | 14.44 | 13.27 | 1.17 |

**Table S2.** DOACs utilization in Hungary between 2014 and 2022 by age group among males and females expressed as defined daily dose per thousand inhabitants per day (DID)

| Age Group/Year | DID 2014 – 2022 (Male) | Coeff. (Male) | p value (Male) | DID 2014 – 2022 (Female) | Coeff. (Female) | p value (Female) |
|---|---|---|---|---|---|---|
| 0 -19 | 0.01- 0.11 | 0.01 | < 0.001 | 0.05 - 0.09 | 0.00 | < 0.005 |
| 20 -24 | 0.11 - 0.44 | 0.05 | < 0.001 | 0.13 - 0.54 | 0.06 | < 0.001 |
| 25 -29 | 0.14 - 0.79 | 0.08 | < 0.001 | 0.24 - 0.84 | 0.08 | < 0.001 |
| 30 -34 | 0.17 -1.56 | 0.18 | < 0.001 | 0.25 - 1.21 | 0.13 | < 0.001 |
| 35 -39 | 0.30 - 2.36 | 0.26 | < 0.001 | 0.29 - 1.83 | 0.21 | < 0.001 |
| 40 - 44 | 0.40 - 3.35 | 0.40 | < 0.001 | 0.37 - 2.63 | 0.29 | < 0.001 |
| 45 - 49 | 0.54 - 5.82 | 0.70 | < 0.001 | 0.52 - 3.75 | 0.43 | < 0.001 |
| 50 - 54 | 0.77 - 8.93 | 1.09 | < 0.001 | 0.56 - 4.99 | 0.60 | < 0.001 |
| 55 - 59 | 1.19 - 14.77 | 1.77 | < 0.001 | 0.67 - 7.59 | 0.90 | < 0.001 |
| 60 - 64 | 1.88 - 24.03 | 2.93 | < 0.001 | 1.16 - 11.90 | 1.41 | < 0.001 |
| 65 - 69 | 3.36 - 40.02 | 4.76 | < 0.001 | 1.96 - 21.29 | 2.52 | < 0.001 |
| 70 - 74 | 4.07 - 59.20 | 7.23 | < 0.001 | 2.86 - 37.23 | 4.54 | < 0.001 |
| 75 -79 | 4.79 - 85.09 | 10.47 | < 0.001 | 3.86 -58.52 | 7.16 | < 0.001 |
| 80 - 84 | 6.31 - 96.73 | 11.77 | < 0.001 | 4.52 - 71.23 | 8.79 | < 0.001 |
| ≥85 | 6.90 - 90.99 | 11.03 | < 0.001 | 4.79 - 69.45 | 8.50 | < 0.001 |

**Table S3** Yearly VKAs consumption by gender in Hungary, 2014 - 2022 (DID):

| Year | Male (DID) | Female (DID) | Difference (Male - Female) |
|---|---|---|---|
| 2014 | 9.55 | 8.12 | 1.43 |
| 2015 | 9.51 | 7.97 | 1.54 |
| 2016 | 9.38 | 7.74 | 1.64 |
| 2017 | 8.97 | 7.27 | 1.70 |
| 2018 | 8.52 | 6.79 | 1.73 |
| 2019 | 7.95 | 6.20 | 1.75 |
| 2020 | 7.45 | 5.73 | 1.72 |
| 2021 | 6.38 | 4.88 | 1.50 |
| 2022 | 5.62 | 4.23 | 1.39 |

**Table S4.** VKAs utilization in Hungary between 2014 and 2022 by age group among males and females expressed as defined daily dose per thousand inhabitants per day (DID)

| **Age Group/Year** | **DID 2014 – 2022 (Male)** | **Coeff. (Male)** | **p value (Male)** | **DID 2014 – 2022 (Female)** | **Coeff. (Female)** | **p value (Female)** |
|---|---|---|---|---|---|---|
| 0 - 19 | 0.15 - 0.12 | 0.00 | < 0.001 | 0.12 - 0.07 | -0.01 | < 0.001 |
| 20 - 24 | 0.35 - 0.17 | -0.02 | < 0.001 | 0.43 - 0.15 | -0.04 | < 0.001 |
| 25 - 29 | 0.71 - 0.37 | -0.04 | < 0.001 | 0.85 - 0.24 | -0.07 | < 0.001 |
| 30 - 34 | 1.24 - 0.59 | -0.08 | < 0.001 | 1.12 - 0.50 | -0.08 | < 0.001 |
| 35 - 39 | 1.92 - 1.03 | -0.11 | < 0.001 | 1.74 - 0.79 | -0.11 | < 0.001 |
| 40 - 44 | 3.22 - 1.68 | -0.20 | < 0.001 | 2.59 -1.22 | -0.18 | < 0.001 |
| 45 - 49 | 5.03 - 2.90 | -0.27 | < 0.001 | 3.71 - 1.83 | -0.24 | < 0.001 |
| 50 - 54 | 8.15 - 4.34 | -0.50 | < 0.001 | 5.07 - 2.59 | -0.30 | < 0.001 |
| 55 - 59 | 13.30 - 7.32 | -0.77 | < 0.001 | 7.23 - 3.69 | -0.46 | < 0.001 |
| 60 - 64 | 20.48 - 11.00 | -1.22 | < 0.001 | 11.04 - 5.54 | -0.69 | < 0.001 |
| 65 - 69 | 30.28 - 15.91 | -1.84 | < 0.001 | 16.88 - 8.14 | -1.12 | < 0.001 |
| 70 - 74 | 41.33 - 22.19 | -2.39 | < 0.001 | 25.24 - 11.33 | -1.80 | < 0.001 |
| 75 - 79 | 51.39 - 27.27 | -3.04 | < 0.001 | 33.53 - 15.88 | -2.28 | < 0.001 |
| 80 - 84 | 57.70 - 32.16 | -3.21 | < 0.001 | 37.95 - 18.51 | -2.57 | < 0.001 |
| ≥85 | 49.51 - 28.65 | -2.66 | < 0.001 | 31.42 - 17.10 | -1.80 | < 0.001 |
